# Alkynyl-Containing Peptides of Marine Origin: A Review

**DOI:** 10.3390/md14110216

**Published:** 2016-11-23

**Authors:** Qiu-Ye Chai, Zhen Yang, Hou-Wen Lin, Bing-Nan Han

**Affiliations:** 1 Research Center for Marine Drugs, Department of Pharmacy, State Key Laboratory of Oncogenes and Related Genes, Renji Hospital, School of Medicine, Shanghai Jiao Tong University, Shanghai 200127, China; chaiqiuye123@sina.cn; 2 School of Pharmacy, Jiangxi University of Traditional Chinese Medicine, Nanchang 330000, China; 3 Department of Pharmacy, Graduate School, Hunan University of Chinese Medicine, Changsha 410208, China; zyyangzhen1991@163.com

**Keywords:** marine cyanobacteria, mollusk, alkynyl peptides, biological activity, absolute configuration

## Abstract

Since the 1990s, a number of terminal alkynyl residue-containing cyclic/acyclic peptides have been identified from marine organisms, especially cyanobacteria and marine mollusks. This review has presented 66 peptides, which covers over 90% marine peptides with terminal alkynyl fatty acyl units. In fact, more than 90% of these peptides described in the literature are of cyanobacterial origin. Interestingly, all the linear peptides featured with terminal alkyne were solely discovered from marine cyanobacteria. The objective of this article is to provide an overview on the types, structural characterization of these unusual terminal alkynyl fatty acyl units, as well as the sources and biological functions of their composed peptides. Many of these peptides have a variety of biological activities, including antitumor, antibacterial, antimalarial, etc. Further, we have also discussed the evident biosynthetic origin responsible for formation of terminal alkynes of natural PKS (polyketide synthase)/NRPS (nonribosome peptide synthetase) hybrids.

## 1. Introduction

As oceans comprise over 70% of the earth’s surface and harbor a tremendous variety of flora and fauna, marine habitat represents a rich source of diverse chemical structures and biological activities of natural products [1], which include alkaloids, terpenoids, peptides, polyketides, steroids, etc. Peptides as an important bioactive natural product, present in many marine species, including sponges, ascidians, seaweeds, mollusks, and marine microorganisms, have been extensively studied [2,3]. Interestingly, diverse structural classes of peptides such as linear peptides, linear depsipeptides, linear lipopeptides, cyclic peptides, cyclic depsipeptides, and cyclic lipopeptides have been discovered from all of these marine species. The broad bioactivity spectrum of marine peptides has high medicinal potential which attracts the attention of the pharmaceutical industry. Since the discovery of the first marine-derived antitumorcyclic peptide, ulithiacyclamide, many marine anticancerpeptides have entered into clinical trials with good prospects for drug development [4,5,6], such as kahalalide F, hemiasterlin, dolastatins, cemadotin, soblidotin, didemnins, aplidine, etc. [7]. Cyclic peptides as a valuable lead for drug discovery with better resistance to enzymatic degradation and higher bioavailability in vivo have attracted considerable attention for further study in the areas of marine natural products [4,8]. Acyclic peptides with the prospect of pharmacological activity are also promising, such as the well-known anticancer lead dolastatin 10 isolated from both sea hare *Dollabella auricularia* [9] and its diet of marine cyanobacterium, the *Symploca* species [10], whose synthetic derivatives have been used in clinical phase III trials [7]. In recent years, a number of structurally intriguing peptides containing diverse fatty acyl units with a terminal alkyne functional group have been found in multiple marine organisms [11,12,13,14], especially marine cyanobacteria and mollusks. The structural characteristics of these peptides with various unusual amino acid residues have displayed their variety of biological functions as antitumor, antibacterial, antimalarial activities, etc., which seemed in some cases correlated to the presence of the terminal alkynyl moieties [14,15,16]. Cyanobacteria, also known as blue-green algae, are ancient photosynthetic prokaryotes living in a wide range of habitats including open oceans, tropical reefs, shallow water environments, and terrestrial substrates. The rich elaboration of biologically active natural products has assisted some of these organisms to survive in predator-rich ecosystems. A major part of cyanobacterial secondary metabolites arepeptides or possess peptidic substructures, which contribute to the more than 600 cyanobacterial peptides discovered thus far [17,18]. Mollusks are the largest marine phylum, comprising about 23% of all the named marine organisms. The gastropods (snails and slugs) are by far the most numerous mollusks in terms of classified species, and account for 80% of the total [19]. To date, over 100 mollusks peptides with diverse structures have been reported (Data based on reviewing the literatures, Marine Natrual Products in Natural Product Reports published during 1985–2015), some of which displayed a variety of bioactivities as antitumor, anti-HIV, ion blockers, etc. [20,21].

In this review, we have provided an overview of the types and structural characterization of these unusual terminal alkynyl fatty acyl units, as well as the sources and biological functions of their composed peptides from marine cyanobacteria and mollusks. Further, we have also discussed the evident biosynthetic origins responsible for formation of terminal alkynes of natural PKS (polyketide synthase)/NRPS (nonribosome peptide synthetase) hybrids, providing perspective insight for drug discovery research.

## 2. Cyclic Peptides Containing Terminal Alkyne

**Figure 1:**
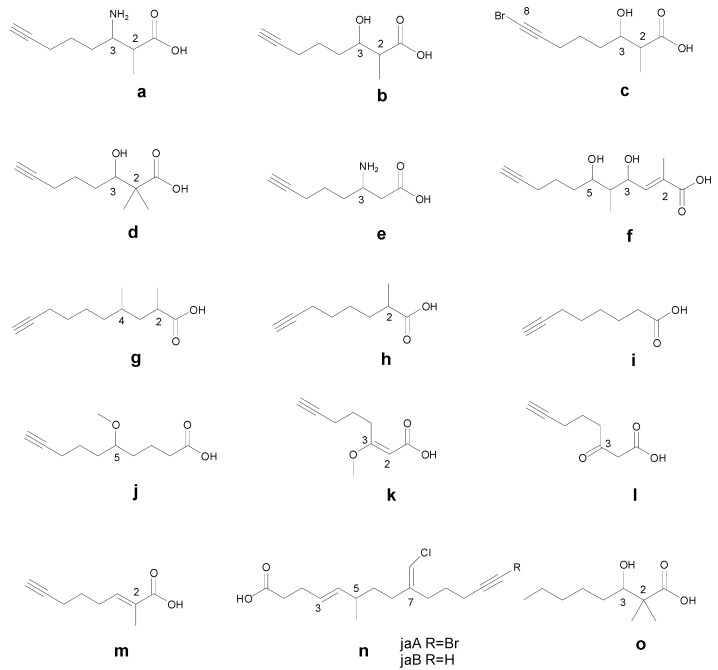
Structures of the terminal alkynyl fatty acyl moieties identified in cyclic/acyclic marine peptides. **a**. 3-amino-2-methyl-7-octynoicacid (Amoya); **b**. 3-hydroxy-2-methyloct-7-ynoic acid (Hmoya); **c**. bromine-containing 3-hydroxy-2-methyloct-7-ynoic acid (Br-Hmoya); **d**. 2,2-dimethyl-3-hydroxy-7-octynoic acid (Dhoya); **e**. 3-amino-6-octyneoic acid (Aoy); **f**. 5,7-dihydroxy-2,6-dimethyldodec-2-en-11-ynoic acid (Dddd); **g**. 2,4-dimethyl-9-decynoic acid; **h**. 2-methyl-7-octynoic acid (Moya); **i**. 7-octynoic acid (Oya); **j**. 5-methoxydec-9-ynoic acid (Mdyna); **k**. 3-methoxy-2-en-7-octynoic acid; **l**. 3-keto-7-octynoic acid; **m**. (*E*)-2-methyloct-2-en-7-ynoic acid; **n**. (4*E*,9*E*)-9-(chloromethylene)-6-methyltetradec-4-en-13-ynoic acid; **o**. 2,2-dimethyl-3-hydroxy-7-octanoic acid (Dhoaa).

A number of terminal alkynylfatty acyl moieties are identified in the cyclic/acyclic marine peptides, which are different by structure and bioactivities (Table 1, Figure 1). Onchidin as the first terminal alkynyl-containing cyclic peptide, featured with 3-amino-2-methyl-7-octynoicacid (Amoya, **a**) moiety was isolated as a molluscan metabolite in 1994 [11]. Since then, Amoya as a component of cyclic peptides has been identified from many marine cyanobacterial metabolites including ulongapeptin, guineamide C, and companeramides A and B. It is likely that the 3-hydroxy-2-methyloct-7-ynoic acid (Hmoya, **b**) moiety was originally discovered in onchidin B from a marine mollusk, and subsequently identified in many cyanobacterial metabolites such as antanapeptin A and D, trungapeptin A, and hantupeptin A. Interestingly, abromine-containing 3-hydroxy-2-methyloct-7-ynoic acidmoiety (Br-Hmoya, **c**) was subsequently identified in several veraguamides isolated from marine cyanobacteria as well. The 2,2-dimethyl-3-hydroxy-7-octynoic acid (Dhoya, **d**) moiety was first discovered as a fatty acyl component in kulolide-1, from a cephalaspidean mollusk, *Philinopsis speciosa*, thereafter reported in many cyclic peptides with cyanobacteria origin as yanucamides A and B, pitipeptolide A, viequeamides A, and more. The 3-amino-6-octyneoic acid (Aoy, **e**) residue and the 5,7-dihydroxy-2,6-dimethyldodec-2-en-11-ynoic acid (Dddd, **f**) residue have been only identified in dolastatin 17 from a marine mollusk *Dolebella auricularia* and in Palau’amide from a marine cyanobacteria *Lyngbya* sp., respectively.

### 2.1. Cyclic Peptides with Dhoya Unit from Marine Cyanobacteria

**Figure 2:**
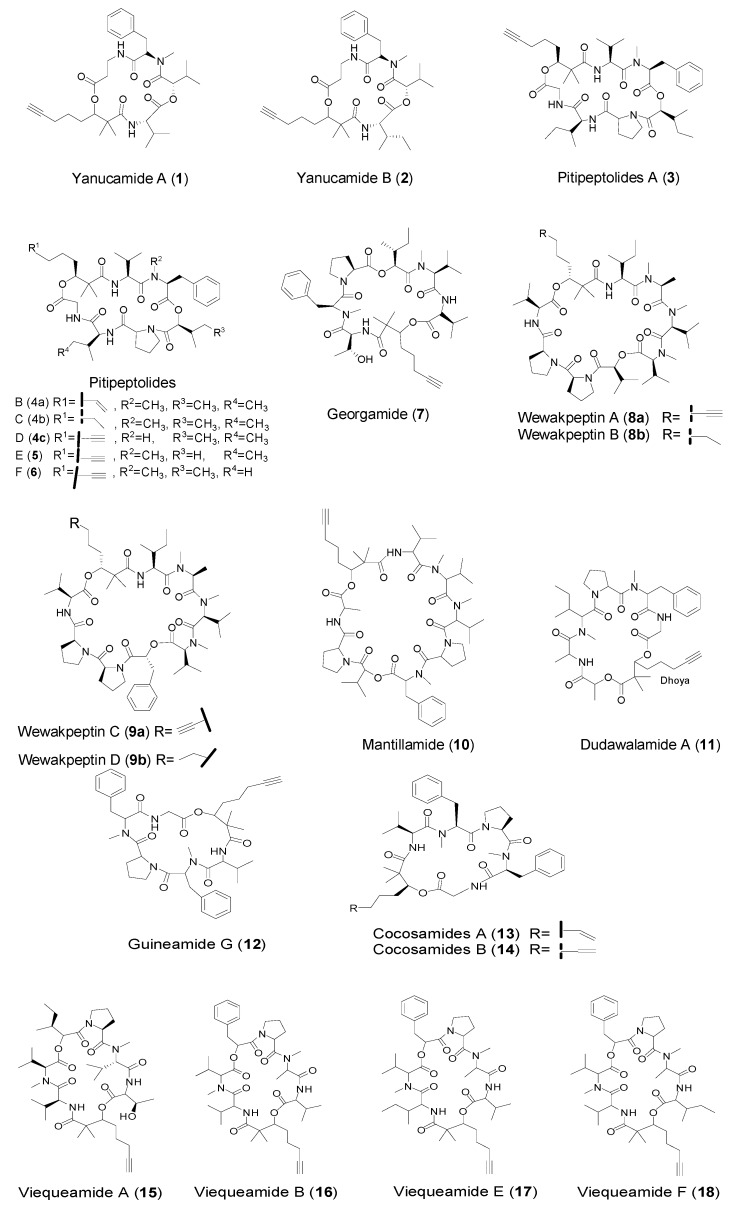
Structures of cyclic peptides with Dhoya residue from marine cyanobacteria.

Cyclic peptides are representative secondary metabolites of cyanobacteria, and in recent years a number of structurally diverse terminal alkynyl-containing cyclic peptides have been found in marine cyanobacteria. The 2,2-dimethyl-3-hydroxy-7-octynoic acid (Dhoya) moiety appeared to be most frequently identified in the terminal alkynyl-containing cyclic peptides. The first two Dhoya unit-containing cyanobacterial cyclic depsipeptides, yanucamides A (**1**) and B (**2**, Table 1, Figure 2), were isolated from the lipid extract of a *Lyngbya majuscula* and *Schizothrix* sp. assemblage collected at Yanuca Island, Fiji, in 2000 [12]. Interestingly, the Dhoya unit had previously been found only in kulolide-1 (**38**) and kulokainalide-1 (**39**), metabolites isolated from the marine mollusk *Philinopsis speciosa*. Thus, the discovery of the yanucamides from a field-collected marine cyanobacterium substantiated the hypothesis that marine cyanobacteria are the probable source of the kulolides and their related metabolites. Both yanucamides A and B displayed strong brine shrimp toxicity (LD_50_, 5 ppm). In 2001, Luesch et al. reported isolation and identification of two new cyclic depsipeptides, pitipeptolides A (**3**, Figure 2) and B, from a population of the marine cyanobacterium *Lyngbya majuscula* collected at Piti Bomb Holes, Guam [22]. Pitipeptolide A with a Dhoya unit and B with a reduced form of Dhoya unit, both showed potent in vitro cytotoxicity against LoVo cells with IC_50_ values of 2.25 and 1.95 μg/mL, respectively; and also exhibited certain growth inhibition for *Mycobacterium tuberculosis* strains ATCC 25177 and ATCC 35818 in the diffusion susceptibility assay. Both compounds were also observed to increase elastase activity (2.76-fold and 2.55-fold, respectively, at 50 μg/mL). Further, in 2011, Luesch et al. revisited larger collections of the same cyanobacterium and obtained additional analogs of pitipeptolides A and B, as well aspitipeptolides C (tetrahydro analog of **3**) and D–F (**4c**, **5**–**6**, Figure 2) [23]. Pitipeptolide A as the major metabolite in this series was reported to act as a feeding deterrent at natural concentrations against a range of marine grazers, suggesting that pitipeptolide A may play an important ecological role among these organisms [54]. Although pitipeptolides C–F were less potent than pitipeptolides A and B against HT-29 colon adenocarcinoma and MCF7 breast cancer cell lines, pitipeptolides C and E showed similar antimycobacterial activities comparable to pitipeptolides A and B. Among them, pitipeptolide F exhibited the highest potency, but pitipeptolide D did not show activities against both mammalian and bacterial cells. As a result, it indicates that the activities of pitipeptolides are not strongly impacted by the Dhoya unit in the structure. Georgamide (**7**, Figure 2), another analog of pitipeptolides featuring Dhoya residue, was obtained from an Australian cyanobacterium Q66C5927 at the head of the King George River, Northwestern Australia [24].

In 2005, an assay-based screening program for anticancer compounds from the marine cyanobacterium *Lyngbya semiplena* collected from Papua New Guinea led to the discovery of four new depsipeptides: wewakpeptins A–D featured with Dhoya or its fully reduced form (Dhoaa, **o**) residues [31]. Intriguingly, wewakpeptins A (**8a**, Figure 2) and B were approximately 10-fold more toxic than C (**9**) and D, with an LC_50_ of approximately 0.4 μM to NCI H-460 human lung tumor and mouse neuroblastoma cells. These cyclic depsipeptides most likely derive from a nonribosomal polypeptide synthetase (NRPS) pathway, and thus, the structural variation of wewakpeptins is intriguing and might suggest that adenylation domains with relaxed substrate specificity are involved in their biosynthesis [31]. Mantillamide (**10**), and dudawalamide A (**11**) featured with Dhoya residues were obtained from the marine cyanobacterium *Lyngbya* sp. because of their biological activity to cancer cells or malaria parasites, and they were able to be identified in a rapid manner using an annotation program developed from tandem mass spectra called MS-CPA available as a web tool (http://lol.ucsd.edu/ms-cpa_v1/Input.py) [25]. Isolation of a new cyclic depsipeptide, guineamide G (**12**) was reported in 2011 from the marine cyanobacterium *Lyngbya majuscula*, collected from Papua New Guinea. Guineamide G was the only cyclic depsipetide featuring Dhoya residue in the series of guineamides, which showed potent brine shrimp toxicity and moderate cytotoxicity to a mouse neuroblastoma cell line with LC_50_ value of 2.7 μM [26]. In 2011, Paul et al. reported isolation and identification of cocosamides A (**13**) and B (**14**) from the lipophilic extract of a collection of *Lyngbya majuscula* from Cocos Lagoon, Guam [27]. Cocosamide A consisting of Dhoea (a reduced form of Dhoya residue) was less potent than cocosamide B (featuring Dhoyaresidue) against HT-29 cells with IC_50_ values of 24 and 11 μM, respectively, indicating the presence of Dhoya moiety may have a slight effect on the cytotoxicity. In 2012, the family of viequeamides A–F was discovered from a shallow subtidal collection of a cyanobacterium (*Rivularia* sp.) near the island of Vieques, Puerto Rico, among which viequeamides A–B (**15**–**16**) and E–F (**17**–**18**, Figure 2) are 2,2-dimethyl-3-hydroxy-7-octynoic acid (Dhoya)-containing cyclic depsipeptides [28]. Intriguingly, viequeamide A was found to be the most active (IC_50_= 60 ± 10 nM) against H460 human lung cancer cell line, whereas the other viequeamides with quite similar structures were inactive.

### 2.2. Cyclic Peptides with Amoya Unit from Marine Cyanobacteria

**Figure 3:**
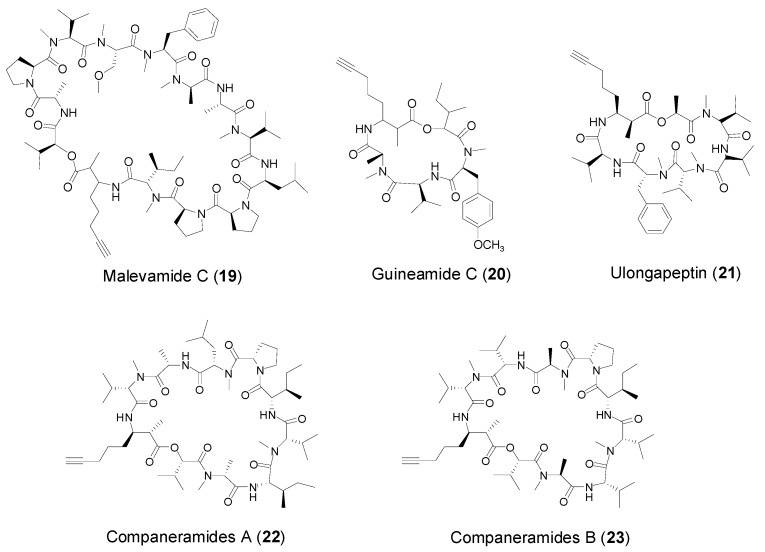
Structures of cyclic peptides with Amoya residue from marine cyanobacteria.

Malevamide C (**19**, Table 1, Figure 3), as the first reported 3-amino-2-methyl-7-octynoic acid (Amoya)-containing cyanobactrial peptide, was obtained from a cyanobactrium *Symplocalaete-viridis* collected in waters adjacent to AlaMoana Beach Park, Hawaii in 2000. The unusual *β*-amino acid residue, Amoya, was only previously identified in onchidin, a cyclic depsipeptide isolated from a marine mollusk *Onchidium* spp. [32]. However, malevamide C did not display potent cytotoxicity against a variety of cancer cell lines. In 2003, another Amoya-containing cyclic depsipeptide, guineamide C (**20**, Figure 3) was discovered by William Gerwick’s group from a Papua New Guinea collection of the marine cyanobacterium *Lyngbya majuscula*. As malevamide C, guineamide C, only exhibited moderate cytotoxicity against neuroblastoma cells with an IC_50_ value of 16 μM [33]. Meanwhile, Williams et al. reported discovery of ulongapeptin (**21**) featuring Amoya residue, isolated from a dark reddish-black clump of cyanobacterium, designated VP755 collected at Ulong Channel in Palau. Interestingly, ulongapeptin showed strong cytotoxicity against KB cells at an IC_50_ value of 0.63 μM [34]. Just recently, two new cyclic depsipeptides, companeramides A (**22**) and B (**23**) containing Amoya unit, were obtained from a marine cyanobacterial assemblage comprising a small filament *Leptolyngbya* species, from Coiba Island, Panama. It is interesting to note that companeramides A and B showed high nanomolar in vitro antiplasmodial activity, though not quite cytotoxic to human cancer cell lines [35].

### 2.3. Cyclic Peptides with Hmoya/Br-Hmoya/Dddd Units from Marine Cyanobacteria

**Figure 4:**
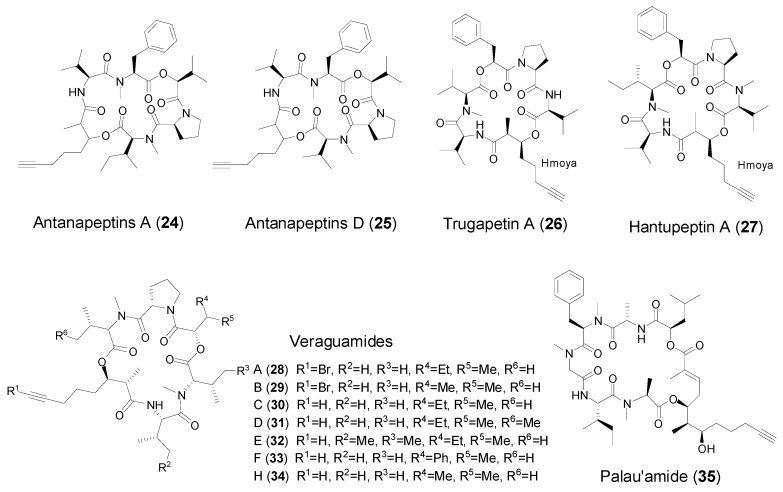
Structures of cyclic peptides with Hmoya/Br-Hmoya/Dddd residue from marine cyanobacteria.

While the 3-hydroxy-2-methyloctynoic acid (Hmoya) residue was initially identified in the molluscan metabolite onchidin B [11,36], antanapeptin A (**24**) and antanapeptin D (**25**, Figure 4) are the first two cyclic peptides containing Hmoya residue, obtained from a cyanobacterium *Lyngbya majuscule* collected from Antany Mora, Madagascar [37]. The antanapeptins were observed inactive in brine shrimp toxicity, sodium channel modulation, and antimicrobial bioassays. Subsequently, Sitachitta et al. in 2006, reported isolation and identification of three new cyclic peptides, trungapeptins A (**26**)–C, containing Hmoya residue, 3-hydroxy-2-methyl-7-octenoic acid (Hmoea), and 3-hydroxy-2-methyl-7-octanoic acid (Hmoaa) residues, respectively [38]. The relative stereochemistry of Hmoya residue of trungapeptin A was determined to be *syn* configuration between H-2 and H-3 by measurement of homonuclear coupling constant as well as comparison of the literature value. The absolute stereochemistry of the Hmoya unit was established as 2*S*, 3*R* by Mosher’s analysis. Intriguingly, herein the stereochemistry of the Hmoya unit is identical to that of kulomo’opunalides [30], but is diastereomeric to that of onchidin B (2*R*, 3*R*). Unlike antanapeptins, trungapeptin A exhibited potent brine shrimp toxicity and ichthyotoxicity at 10 ppm and 6.25 ppm, respectively. However, it was inactive against KB and LoVo cells at 10 μg/mL. In 2009, a new Hmoya-containing analog of trungapeptin A, hantupeptin A (**27**, Figure 4) was discovered from the marine cyanobacterium *Lyngbya majuscula* from PulauHantuBesar, Singapore [39]. The absolute configuration at C-3 was determined to be *S* by Mosher’s analysis following methanolysis of hantupeptin A and isolation of the Hmoya fragment. However, the stereochemistry at C-3 of the Hmoya unit in hantupeptin A is different from that of trungapeptin A. Further, hantupeptin A afforded both brine shrimp toxicity at 10 ppm and strong cytotoxicity against the leukemia cell line MOLT-4 with an IC_50_ value of 32 nM.

In 2011, the Luesch group and Gerwick group coincidently reported isolation and identification of a series of peptides featured with Hmoya and its derived residues, veraguamides A–F (**28**–**33**), from a cyanobacterium *Symploca* cf. *hydnoides* at Cetti Bay, Guam [40], and veraguamides H (**34**), I–L from the marine cyanobacterium cf. *Oscillatoria margaritifera* at the Coiba National Park, Panama [13], respectively. Among them, veraguamides A and B are 8-bromo-3-hydroxy-2-methyl-7-octynoic acid (Br-Hmoya) moiety-containing cyclic peptides, while veraguamides K and L (**63**–**64**) are Br-Hmoya-containing linear peptides (more in Section 3). It is interesting to note that veraguamides D and E were five-fold more potent than their related congener veraguamide C against HT29 colorectal and HeLa cervical adenocarcinoma cells, while veraguamides A, B and F were inactive againstthese cancer cell lines. Surprisingly, veraguamide A exhibited strong potency in the H-460 cytotoxicity assay (LD_50_ = 141 nM), but veraguamides B, C, K and L were much less active.

Palau’amide (**35**, Figure 4) is a unique terminal alkynyl-containing cyclic depsipeptide, consisting of a novel polyketide unit, 5,7-dihydroxy-2,6-dimethyldodec-2-en-11-ynoic acid (Dddd), which was obtained from a *Lyngbya* sp. from Palau. Palau’amide showed strong cytotoxicity against KB cells with an IC_50_ value of 13 nM [41].

### 2.4. Cyclic Peptides from Marine Mollusks

**Figure 5:**
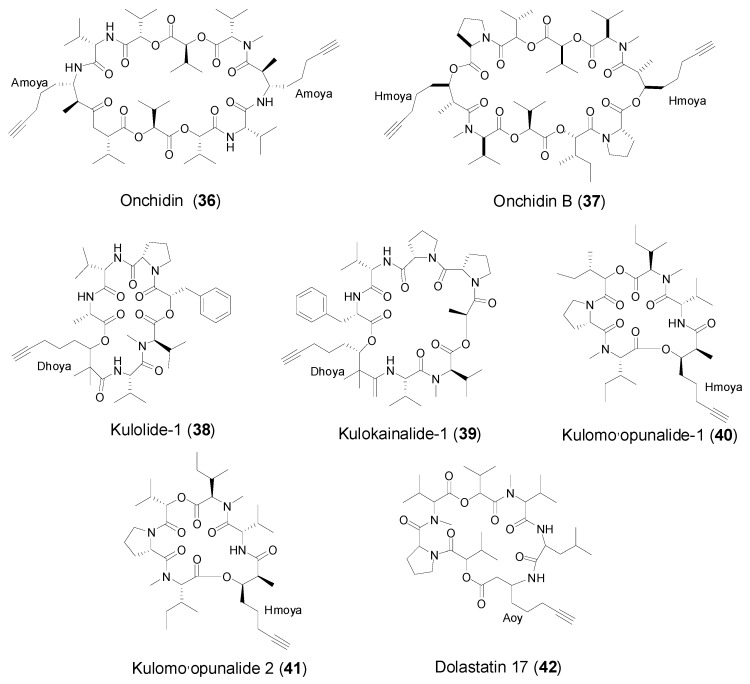
Structures of cyclic peptides with Amoya/Hmoya/Dhoya/Aoy residue from marine mollusks.

Onchidin (**36**, Figure 5) as the first report of a dimeric depsipeptide from a mollusc, featured with two 3-amino-2-methyl-7-octynoicacid (Amoya, **a**) residues, was obtained from the pulmonate mollusk *Onchidium* sp. collected off New Caledonian 1994 [11]. Onchidin B (**37**) isolated and identified along with onchidin from the same extract, shares quite similar structural features with onchidin. Interestingly, onchidin B featured with two 3-hydroxy-2-methyloct-7-ynoic acid (Hmoya, **b**) does not have a *C*_2_ axis of symmetry as does onchidin, due to the presence of the two enantiomers of proline that renders the two halves of the molecule different [36]. Onchidin and onchidin B exhibited identical cytotoxicity against P-388 murine leukemia cells (IC_50_ = 8 μg/mL) and Kb human epidermoid carcinoma cells (IC_50_ = 8 μg/mL), respectively.

A cephalaspidean mollusk, *Philinopsis speciosa* Pease, 1860 collected off North Shore, Oahu’s (Hawaiian Islands) Shark Bay, afforded the first 2,2-dimethyl-3-hydroxy-7-octynoic acid (Dhoya)-containing cyclic depsipeptide, kulolide-1 (**38**, Figure 5) [29]. Kulolide-1was active against L-1210 leukemia cells and P388 murine leukemia cells at IC_50_ values of 0.7 and 2.1 μg/mL, respectively. Along with kulolide-1, three other terminal alkynyl-containing cyclic depsipeptides, kulokainalide-1 (Dhoya, **39**), kulomo’opunalide-1 (Hmoya, **40**) and kulomo’opunalide-2 (Hmoya, **41**), were also discovered from the same sample of the cephalaspidean mollusk, *Philinopsis speciosa* [30].

3-amino-6-octyneoic acid (Aoy, **e**) as an unprecedented terminal alkynyl moiety, was only identified in a novel cyclic depsipeptide, dolastatin 17, isolated from a sea hare *Dolebella auricularia* [12]. Dolastatin 17 (**42**, Figure 5) displayed significant growth-inhibitory activity against OVCAR-3 (GI50 0.67 μg/mL), SF-295 (GI50 0.55 μg/mL), NCI-H460 (GI50 0.74 μg/mL), KM20L (GI50 0.45 μg/mL) human cancer cell lines [42].

## 3. Acyclic Lipopeptides Containing Terminal Alkyne from Marine Cyanobacteria

It is interesting to note that many linear peptides have also been found to possess the terminal alkynyl fatty acyl moieties, including 2,4-dimethyl-9-decynoic acid (**g**), 2-methyl-7-octynoic acid (Moya, **h**), 7-octynoic acid unit (Oya, **i**), 5-methoxydec-9-ynoic acid (Mdyna, **j**), 3-methoxy-2-en-7-octynoic acid (MeO-Oya-2-ene, **k**), 3-keto-7-octynoic acid (**l**), and (*E*)-2-methyloct-2-en-7-ynoic acid (**m**), which are different from that of cyclic peptides, except for Hmoya and Br-Hmoya residues present in both linear and cyclic veraguamides (Table 1). In addition, an acyclic amide-like secondary metabolite from the marine cyanobacteria *Lyngbya majuscula*, termed jamaiapcamides A, has provided an alkynyl bromide, vinyl chloride, *β*-methoxyeneone moiety (**n**) to the terminal alkynyl-containing peptides.

**Figure 6:**
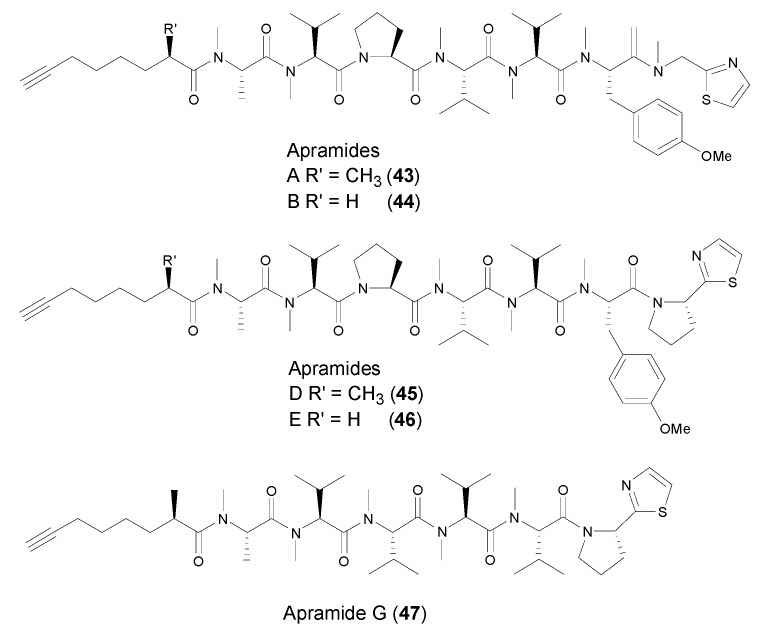
Structures of linear peptides (apramides A–G) from marine cyanobacteria.

All the terminal alkynyl-containing linear peptides were solely discovered from marine cyanobacteria. In 2000, Luesch et al. reported the isolation and identification of six new linear peptides, apramides A–G (Figure 6), from the marine cyanobacterium *Lyngbya majuscule* collected at Apra Harbor, Guam [43]. Apramides A (**43**), D (**45**) and G (**46**) are Moya-containing acylic peptides, while apramides C and F consist of 2-methyl-7-octenoic acid moiety (Moea) in their structures. Apramides B (**44**) and E (**47**) possess a 7-octynoicacid unit (Oya) in lieu of the Moya moiety, and the rest of the structures are identical to apramides A and D, respectively. Apramides A–G was inactive in cytotoxic, antibacterial, antifungal assays, but apramide A exhibited stimulating elastase activity.

**Figure 7:**
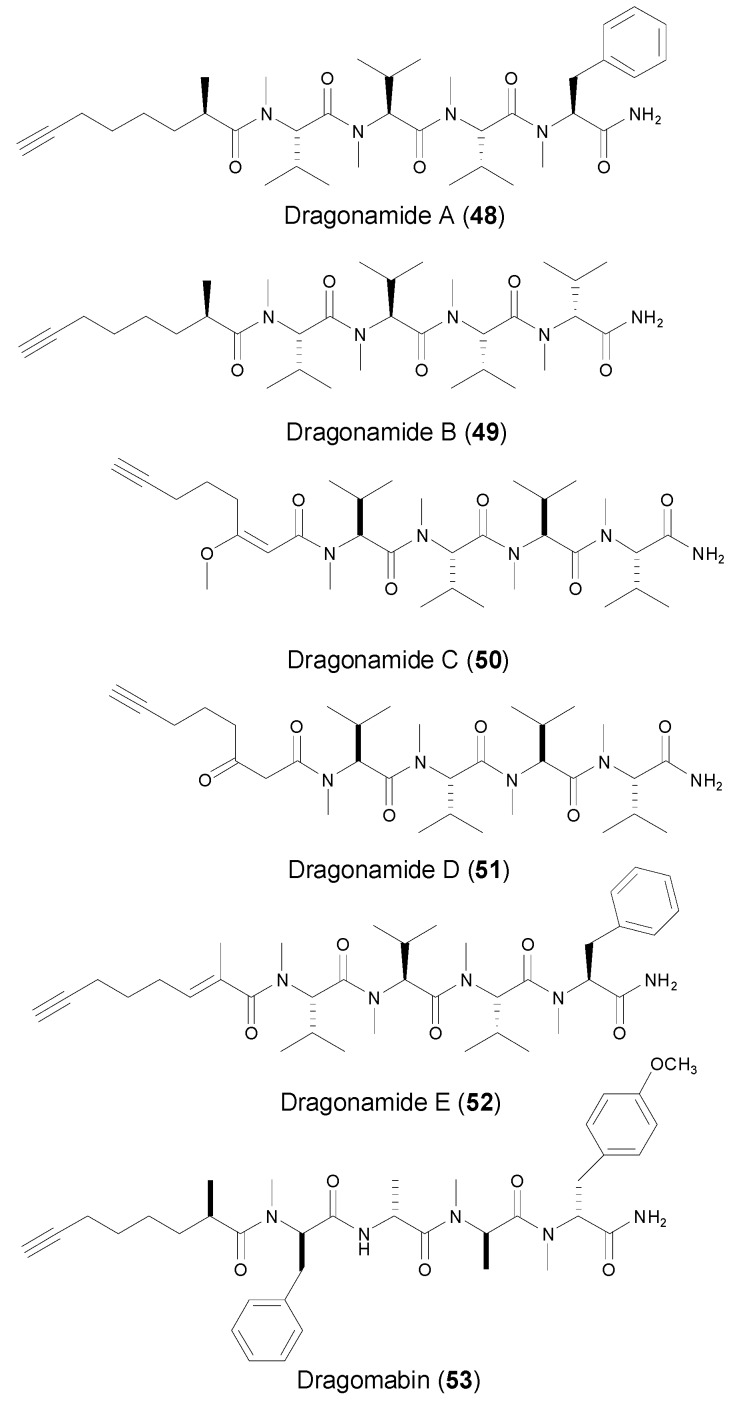
Structures of linear peptides (dragonamides A–E, dragomabin) from marine cyanobacteria.

Dragonamides are a family of structurally close linear peptides composing of a variety of terminal alkynyl units (Figure 7). Several separate Panamanian collections of *Lyngbya majuscule* Gomont afforded dragonamides A, B (**48**–**49**) and E [44,45,46], while the collection of brown *Lyngbya polychroa* from Hollywood Beach, Fort Lauderdale, FL led to the discovery of dragonamides C and D [47]. Dragonamides A and B contain a terminal 2-methyl-7-octynoic acid unit (Moya), whereas dragonamides C, D and E (**50**–**52**) possess three different terminal acetylene units, 3-methoxy-2-en-7-octynoic acid (**k**), 3-keto-7-octynoic (**l**) (*E*)-2-methyloct-2-en-7-ynoic acid (**m**), respectively, which were not previously reported from marine peptides. Dragonamides did not exhibit strong activities against a variety of tumor cell lines, except dragonamides A and E which showed moderate in vitroactivity against leishmaniasis. Along with dragonamides A and B, another terminal Moya-containing linear peptide, dragomabin (**53**, Figure 7), was isolated and identified in 2007, from a Panamanian strain of the marine cyanobacterium *Lyngbya majuscula* [45]. Dragomabin possesses the best differential toxicity between parasite and mammalian cells, with IC_50_ value of 6.0 μM against the W2 chloroquine-resistant malaria strain and IC_50_ value of 182.3 μM against Vero cells (kidney epithelial cells).

**Figure 8:**
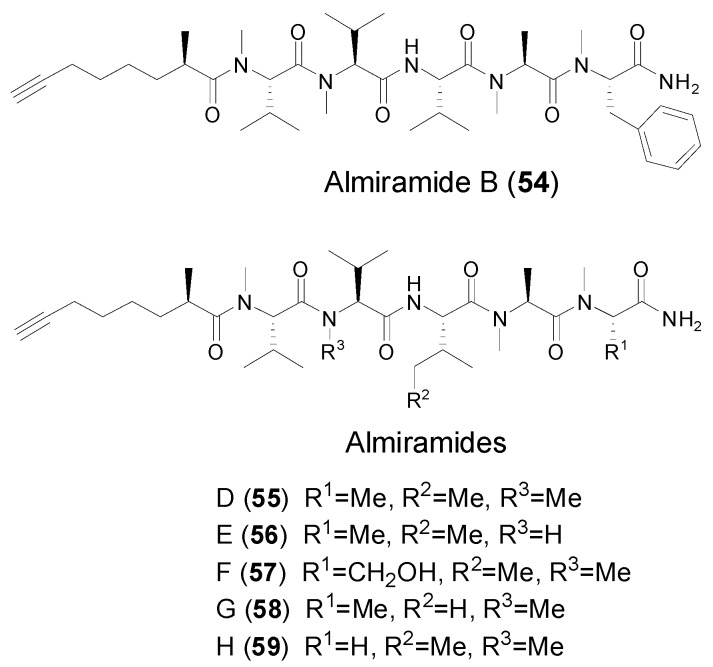
Structures of linear peptides (almiramide B, D–H) from marine cyanobacteria.

In 2010, Linington et al. reported the isolation and identification of a series of terminal fatty acyl units-containing linear peptides, almiramides A–C, from a Panamanian strain of the marine cyanobacterium *Lyngbya majuscule* [14]. Among them, almiramide B (**54**) is featured with a terminal Moya unit (Figure 8), whereas almiramide C contains a reduced form of Moya as a 2-methyloct-7-enoic acid residue. Biological evaluation of these three compounds showed that almiramides B and C possessed good selectivity between parasite and mammalian cells with strong in vitro antiparasitic activity against *leishmania* (IC_50_ = 2.4 and 1.9 μM, respectively), and weak activity against Vero cells (IC_50_ = 52.3 and 33.1 μM, respectively). Just recently, a series of new terminal Moya-containing linear peptides, almiramides D–H (**55**–**59**) along with known almiramide B (Figure 8), were isolated and identified from a cyanobacterium sample of *Oscillatoria nigroviridis* collected at the Colombian Caribbean Sea [48]. Intriguingly, two structurally representative almiramides B and D showed mild toxicity against five human tumor cell lines, but high toxicity against the gingival fibroblast cell line was used as reference to evaluate selectivity against tumor cell lines compared with primary cell line.

**Figure 9:**
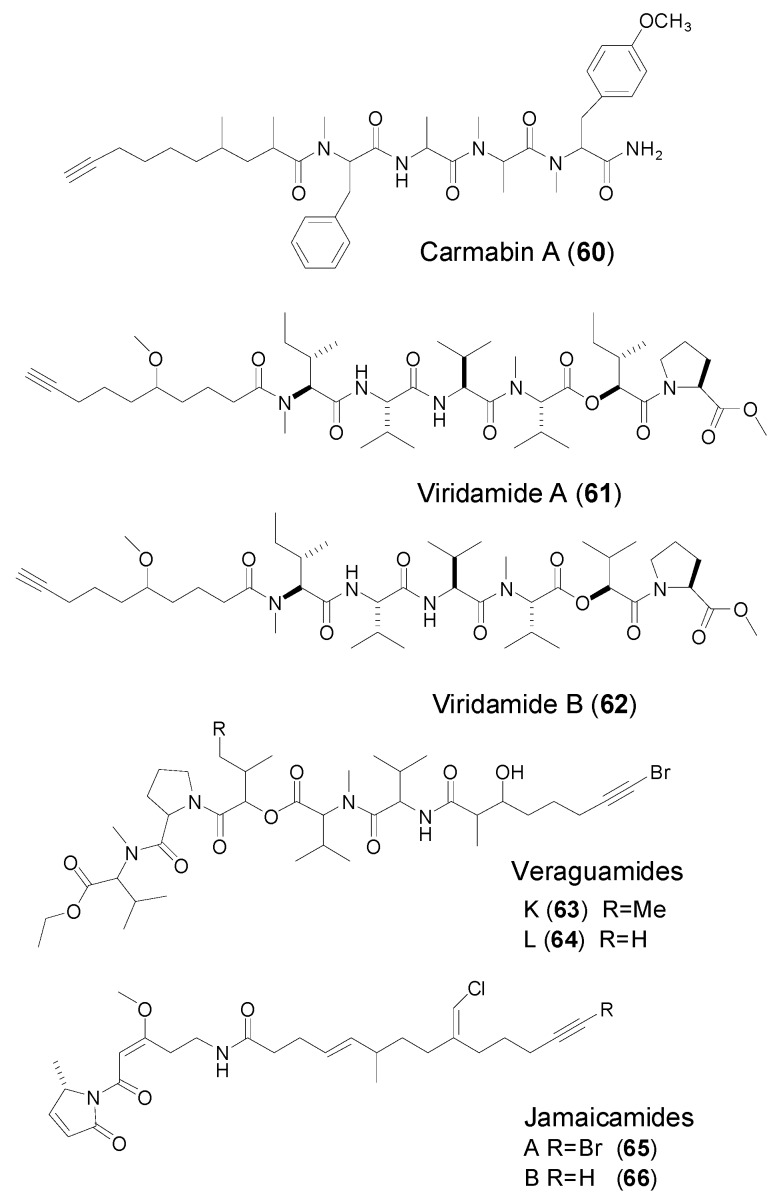
Structures of linear peptides (carmabin A, viridamide A–B, veraguamides K and L, and jamaicamides A–B) from marine cyanobacteria.

Two novel terminal fatty acyl-containing linear peptides, carmabins A (**60**) and B were discovered from a collection of the marine cyanobacterium *Lyngbya majuscule* at Barbara Beach (Spanish Waters), Curacao, Netherlands Antilles in 1998 [50]. Carmabin A (Figure 9) is featured with a novel terminal 2,4-dimethyl-9-decynoic acid residue, but in carmabin B, the acetylene functional group is replaced with a methyl ketone. To the best of our knowledge, carmabin A is the only reported compound containing a 2,4-dimethyldec-9-ynoic acid moiety. Carmabin A exhibited moderate cytotoxicity to Vero cells (IC_50_ = 9.8 μM), and mild activity against the W2 chloroquine-resistant malaria strain (IC_50_ = 4.3μM).

In 2008, Simmons et al. reported discovery of two new linear peptides, viridamides A and B (**61**–**62**, Figure 9) isolated from the marine cyanobacterium *Oscillatoria nogroviridis* [51] (Figure 9), whose structures contain a novel terminal 5-methoxydec-9-ynoic acid moiety (Mdyna). Viridamide A displayed antitrypanosomal activity (IC_50_ 1.1 μM to *Trypanosoma cruzi*) and antileishmanial activity (IC_50_ 1.5 μM to *Leishmania mexicana*).

## 4. Different Methods to Determine the Absolute Configuration of Different Alkynyl Fragments

### 4.1. Amoya (**a**)

Determination of stereochemistry of the 3-amino-2-methyl-7-octynoic acid (Amoya, **a**) residue in the cyclic depsipeptides was established using differential methods such as NMR or Marfey’s analysis. The configuration of an Amoya unit in onchidin was found to be threo through analysis of the NOE data and their coupling constants for critical protons, which indicated the relative stereochemistry of the pentine side chain on the same side as the neighboring MeVal and Val isopropyl groups. As a result, the absolute configuration of an Amoya unit in onchidin was determined to be 7*S*, 9*S* [11]. The stereochemistry of the Amoya unit in ulongapeptin was determined using the synthetically saturated 3-amino-2-methyloctanoic acid C-2 diastereomers (2*R*, 3*R* and 2*S*, 3*R*) as standards for Marfey’s analysis. Comparison with the derivatized hydrogenated hydrolysate of ulongapeptin established the absolute configuration of the Amoya as 2*S*, 3*S* [34]. Surprisingly, the absolute configuration of the Amoya unit in companeramides A (**22**) and B (**23**) was determined to be 2*S*, 3*R* using the method of Marfey’s analysis in comparison with synthetically saturated 3-amino-2-methyloctanoic acid C-2 diastereomeric (2*R*, 3*R* and 2*S*, 3*R*) standards [35].

### 4.2. Hmoya (**b**)

Determination of stereochemistry of 3-hydroxy-2-methyloct-7-ynoic acid (Hmoya) was first accomplished in the work of identification of onchidin B [36]. As beginning of the work, all four possible stereoisomers of Hmoya were synthesized in a diastereo selective mode. However, direct comparative analysis of the methyl esters of the four synthetic standards with the methyl ester of the natural Hmoya hydrolyzed from onchidin B using chiral gas chromatography (GC) and HPLC was not successful due to a separation issue. Consequently, the problem was overcome by derivation of the four hydroxy esters with (−)-(*R*)-α-methoxy-α-(9-anthryl) acetic acid as well as the natural Hmoya component to obtain good resolution of the four synthetic stereoisomers in LC-MS analysis, which indicated that the absolute configuration of Hmoya moiety in onchidin B was 2*R*, 3*R*. The stereochemistry of the Hmoya unit in Kulomo’opunalide-1 (**40**) and kulomo’opunalide-2 (**41**) was initially worked on comparison of chemical shifts of the *p*-bromobenzoyl derivatized synthetic standards with the derivatized natural Hmoaa (hydrogenated form of Hmoya) in ^1^H NMR spectra to provide the relative stereochemistry of 2S*, 3R*. Comparison of retention time and co-injection of the standards with hydrolyte of the hydrogenated (**40**) and (**41**) confirmed the absolute stereochemistry of the Hmoya unit as 2*S*, 3*R* [30], which is surprisingly different from 2*R*, 3*R* of the Hmoya unit in onchidin B. Interestingly, the absolute configuration of the Hmoya unit in trungapeptin A (**26**) was determined to be 2*S*, 3*R* by application of the *J*-based configuration analysis as well as Mosher’s method [38]. Further, the stereochemistry of Hmoya in hantupeptin A (**27**) was determined to be *S* at C-3 using Mosher’s analysis, but the configuration at C-2 was not established [39]. In addition, the absolute configuration of the Br-Hmoya unit in veraguamide A (**28**) was also determined to be 2*S*, 3*R* identical to that of trungapeptin A using the *J*-based configuration analysis as well as the Mosher’s method subjected to the linear veraguamide A following methanolysis of 28 [40].

### 4.3. Dhoya (**d**)

Determination of absolute configuration of 2,2-dimethyl-3-hydroxy-7-octynoic acid (Dhoya) residue was initially achieved in the structure elucidation of kulolide-1 (**38**), which was treated with NaOMe to release the free hydroxyl functional group in the Dhoya-containing fragment, followed by Mosher’s analysis to reveal the *R*-configuration at C-3 of Dhoya [29]. Interestingly, the stereochemistry of the Dhoya unit in kulokainalide-1 was determined to be 3*S* by comparing the values of optical rotation of Dhoaa (saturated form of Dhoya) residues obtained from the acid hydrolysates of both hydrogenated kulolide-1 and kulokainalide-1 [30]. Further, Ye et al. achieved a total synthesis of yanucamide A to confirm the absolute configuration of Dhoya to be the same (3*S*) as in kulokainalide-1 [55]. The stereochemistry of the Dhoya unit in pitipeptolide A (**3**) was also revealed as 3*S* using the optical rotation data of the obtained Dhoaa unit [22]. Interestingly, the absolute configuration of the Dhoya unit in wewakpeptin A (**8**) was determined to be *R* by chiral GC-MS analysis of the hydrogenated Dhoya in **8** possessing the same retention time as synthetic *R*-Dhoaa [31]. The chiral center of Dhoya residue in cocosamide B (**14**), was suggested to possess the same 3*S* configuration as in pitipeptolide A, by comparison of the NOE correlations of specific protons observed for Dhoya as well as related protons in the structures of cocosamide B and pitipeptolide A [27]. The configuration of Dhoya residue in viequeamide A was revealed to be *S* by chiral GC-MS analysis of the synthetic standards and the obtained natural Dhoya unit [28].

### 4.4. Moya (**h**)

The 2-methyl-7-octynoic acid (Moya, **h**) unit is the most frequently identified terminal alkynyl residue in the linear peptides. The absolute configuration at C-2 in apramides was proposed to be *R* based on the negative contribution of the C-2 stereocenter to the molar optical rotation of the molecule [50], because it is known for a closely related model compound that the 2*S* epimer gives a more positive rotation in CHCl_3_ than the corresponding epimer with *R* configuration in the lipid chain [56]. The stereochemistry of Moya residue in dragonamide A was initially determined to be *R*, which was inferred by comparison of optical rotation data of 2-methyloctanoic acid obtained from hydrolyte of hydrogenated dragonamide A with literature values of other 2-methylalkanoic acids [57,58]. Subsequently, the later total synthesis of dragonamide A has led to a reassignment of the configuration as *S* at the stereogenic center of the Moya unit of the molecule [16]. Further, dragonamide B and dragomabin were isolated with dragonamide A from a Panamanian collection of *Lyngbya majuscule* Gomont, while the NMR and optical rotation data for this dragonamide A closely match the 2*S* synthetic product, but differ significantly from the 2*R* synthetic product [45]. Therefore, it was concluded that dragonamide A, dragonamide B, and dragomabin all contain 2*S*-methyloct-7-ynoic acid. The stereochemistry at C-2 of Moya residue in almiramides B and C was investigated by comparison of commercial standards with obtained natural Moya derivatives using GC-MS, which was determined to be *R* configuration [46], surprisingly opposite to the absolute configuration of the Moya unit in dragonamides.

### 4.5. Other Special Fragments

Determination of stereochemistry of 5,7-dihydroxy-2,6-dimethyldodec-2-en-11-ynoic acid (Dddd, **f**) residue in Palau’amide was a bit complex, due to an inter-converting mixture of rotamers around these stereocenters of Dddd. With the secured NMR assignments for the two major conformers of Palau’amidein CDCl_3_ (C-R1/-R2), subsequent NOE experiments recorded in CDCl_3_ revealed a strong correlation between H-40 and H-46 that indicated the *erythro* configuration of C-38 and C-39. The Mosher’s analysis of the absolute configuration of C-39 was carried on the α-methoxy phenyl acetic acid (MPA) derivatives of Palau’amide. Comparison of the Δδ^*RS*^ values for these derivatives established the *R* configuration of C-39 [41]. While the configuration of C-37 could not be rigorously established by chemical means, analysis of molecular models in conjunction with NOE data suggested an *S*-configuration for this chiral center. The double bond configuration of 3-methoxy-2-en-7-octynoic acid (**k**) in dragonamide C and that of 2-methyloct-2-en-7-ynoic acid (**m**) in dragonamide E, were both assigned as E-geometry by NOE analysis [47,48].

## 5. Conclusions

A number of structurally intriguing peptides containing diverse terminal alkynyl fatty acyl residues, such as Dhoya, Hmoya, Amoya, Aoy, Moya, etc., have been found in multiple marine organisms, especially marine mollusk and cyanobacteria. In 1998, a study about the biological origin of Dhoya-containing cyclic depsipeptide, kulolide-1, by Scheuer and coworkers showed that the marine mollusk *Philinopsis speciosa* preyed on the herbivorous sea hare *Stylocheilus longicaudus* that is well recognized to possess the predator-prey relationship with cyanobacteria [30]. Interestingly, Scheuer and coworkers succeeded in isolating kulolide-1 from sea hare *Stylocheilus longicaudus*, which suggests that kulolide-1 discovered from *P. speciosa* is possibly accumulated from its prey *Stylocheilus longicaudus*, known to sequester secondary metabolites from its diet of mat-forming cyanobacteria [29]. Thus, similarity among the terminal alkynyl-containing cyclic peptides is suggestive that this intriguing structure family of metabolites in fact originates in cyanobacteria. Interestingly, all the terminal alkynyl fatty acyl moieties identified in the linear peptides were solely discovered as the constituents of metabolites of marine cyanobacteria.

Overall, many of these terminal alkynyl-containing peptides have shown a variety of biological functions as antitumor, antibacterial and antimalarial activities. Intriguingly, some of them with minor structural variations have presented different biological effects. For example, viequeamide A was found to be the most active (IC_50_ = 60 ± 10 nM) against H460 human lung cancer cell line, whereas the other viequeamides with quite similar structures were inactive; hantupeptin A exhibited strong cytotoxicity against the leukemia cell line MOLT-4 with an IC_50_ value of 32 nM, but trungapeptin A was reported to be inactive against KB or LoVo cells at 10 μg/mL. Some cases further indicated that the unsaturated terminal moieties may play an important role in the biological activity, as illustrated by almiramide B and C possessing strong in vitro antiparasitic activity against *L. donovani*, whereas almiramide A was completely inactive.

Another research area to exploit marine peptides as a source of new therapeutics is to harness the genetic versatility of its biosynthetic gene clusters. Acetylenases, a special family of desaturases that catalyze O_2_-dependent dehydrogenation of C–C bonds, have been considered to be responsible for formation of terminal alkynes of many natural products [15]. In 2015, Zhu and Zhang et al. reported a thorough characterization of terminal alkyne biosynthetic enzymes responsible for the synthesis of jamaicamide A and B (**65**–**66**) and carmabins [51,52], which demonstrated the in vitro formation of a short-chain alkynoic starter unit by a three-gene operon, *jamABC*, where *jamA*, *jamB* and *jamC* encode a homolog of fatty acyl-CoA ligase, a membrane-bound fatty acid desaturase and an acyl carrier protein (ACP), respectively [53]. Therefore, the biosynthetic evidences have further shown that the fatty acyl starter unit and the extender units could be engineered using *jamABC* and other modular assembly lines of PKS/NRPS enzymatic machinery to form the terminal alkyne-containing natural product.

A well-known reaction referred to as the “click reaction” (the triazole forming via azide-alkyne cyclo addition), has been quite often used in selective imaging and study of azide- or alkyne-labeled macromolecule interaction. In our opinion, the azide-alkyne click chemistry may serve as a powerful tool to study the drug mechanism of the terminal alkyne-containing peptides as well as to explore their structure activity relationship (SAR). Not surprisingly, it is highly expected to see application of the “click reaction” in combination with the biosynthetically engineered alkynyl-containing peptides playing a role in drug discovery research in the near future.

## Figures and Tables

**Table 1:** Terminal alkynyl-containing cyclic/acyclic peptides from marine cycanobacteria and mollusks.

Moiety Unit	Compound	Organism	Bioactivities	Reference
Dhoya	Yanucamides A (**1**) and B (**2**)	Marine cyanobacterium *Lyngbya majuscule*, *Schizothrix* sp.	Strong brine shrimp toxicity	[12]
	Pitipeptolides A (**3**) Pitipeptolides D–F (**4**–**6**)	Marine cyanobacterium *Lyngbya majuscula*	Antitumor cytotoxicity	[22,23]
	Georgamide (**7**)	Marine cyanobacterium	anti-HIV cytotoxicity	[24]
	Mantillamide (**10**) Dudawalamide A (**11**)	Marine cyanobacterium *Lyngbya* sp.	Antitumor cytotoxicity Antimalaria parasites	[25]
	Guineamide G (**12**)	Marine cyanobacterium *Lyngbya majuscula*	Brine shrimp toxicity Antitumor cytotoxicity	[26]
	Cocosamides A–B (**13**–**14**)	Marine cyanobacterium *Lyngbya majuscula*	Antitumor cytotoxicity	[27]
	Viequeamides A–B (**15**–**16**) and E–F (**17**–**18**)	Marine cyanobacterium *Rivularia* sp.	Antitumor cytotoxicity	[28]
	Kulolide-1 (**38**)	Marine mollusk *Philinopsis speciosa* Pease	Antitumor cytotoxicity	[29]
	Kulokainalide-1 (**39**)	Marine cephalaspidean mollusk *Philinopsis speciosa*	Moderate antitumor cytotoxicity	[30]
Dhoaa	Wewakpeptins A and C (**8a**–**9**)	Marine cyanobacterium *Lyngbya semiplena*	Antitumor cytotoxicity	[31]
Amoya	Malevamide C (**19**)	Marine cyanobacterium *Symplocalaete-viridis*	No cytotoxicity	[32]
	Guineamide C (**20**)	Marine cyanobacterium *Lyngbya majuscula*	Antitumor cytotoxicity	[33]
	Ulongapeptin (**21**)	Marine cyanobacterium *Lyngbya* sp.	Antitumor cytotoxicity	[34]
	Companeramides A–B (**22**–**23**)	Marine cyanobacterium *Leptolyngbya* sp.	Antiplasmodial activity	[35]
	Onchidin (**36**)	Marine pulmonate mollusk *Onchidium* sp.	Strong antitumor cytotoxicity	[11,36]
Hmoya	Antanapeptin A and D (**24**–**25**)	Marine cyanobacterium *Lyngbya majuscula*	Na^+^ channel modulation Antimicrobial activity	[37]
	Trungapeptins A (**26**)	Marine cyanobacterium *Lyngbya majuscula*	Brine shrimp toxicity and ichthyotoxicity	[30,38]
	Hantupeptin A (**27**)	Marine cyanobacterium *Lyngbya majuscula*	Brine shrimp toxicity Antitumor cytotoxicity	[39]
	Veraguamides B–F (**29**–**33**)	Marine cyanobacterium *Symploca* cf. *hydnoides*	Veraguamides A and C, antitumor cytotoxicity	[40]
	Veraguamides H (**34**)	Marine cyanobacterium *Oscillatoria margaritifera*	No cytotoxicity	[13]
	Onchidin B (**37**)	Marine pulmonate mollusk *Onchidium* sp.	Strong antitumor cytotoxicity	[11,36]
	Kulomo’opunalide-1 (**40**) and (**41**)	Marine cephalaspidean mollusk *Philinopsis speciosa*	Moderate antitumor cytotoxicity	[30]
Dddd	Palau’amide (**35**)	Marine cyanobacterium *Lyngbya* sp.	Strong antitumor cytotoxicity	[41]
Aoy	Dolastatin 17 (**42**)	Marine mollusk *Dolebella auricularia*	Antitumor cytotoxicity	[12,42]
Oya	Apramides B and G (**44**,**47**)	Marine cyanobacterium *Lyngbya majuscula*	Apramide A exhibited stimulating elastase activity	[43]
Moya	Apramides A,D and G (**43**,**45**–**46**)	Marine cyanobacterium *Lyngbya majuscula*	Apramide A exhibited stimulating elastase activity	[43]
	Dragonamides A–B (**48**–**49**)	Marine cyanobacterium *Lyngbya majuscule* Gomont	Antileishmaniasis	[44,45,46,47]
	Dragonamides C–E (**50**–**52**)	Marine cyanobacterium *Lyngbya polychroa*	Antileishmaniasis	[47]
	Dragomabin (**53**)	Marine cyanobacterium *Lyngbya majuscula*	Antiparasite toxicity	[45]
	Almiramide B (**54**)	Marine cyanobacterium *Lyngbya majuscule*	Antitumor cytotoxicity	[14]
	Almiramides D–H (**55**–**59**)	Marine cyanobacterium *Oscillatoria nigroviridis*	Antitumor cytotoxicity	[48]
Mdyna	Viridamides A–B (**61**–**62**)	Marine cyanobacterium *Oscillatoria nigro*-*Wiridis*	Antitrypanosomal activity Antileishmanial activity	[49]
Br-Hmoya	Veraguamides A (**28**)	Marine cyanobacterium *Symploca* cf. *hydnoides*	Veraguamides A and C, antitumor cytotoxicity	[40]
	Viridamides K–L (**63**–**64**)	Marine cyanobacteria, cf. *Oscillatoria margaritifera*	Antitumor cytotoxicity	[13]
2,4-dimethyl-9-decynoic acid	Carmabins A (**60**)	Marine cyanobacterium *Lyngbya majuscula*	Antimalaria against the W2 chloroquine-resistant malaria strain	[50]
9-(chloromethylene)-6-methyltetradec-4-en-13-ynoic acid	Jamaicamide A–B (**65**–**66**)	Marine Cyanobacterium *Lyngbya majuscula*	not mentioned	[51,52,53]
